# *Egr*-1 induction provides a genetic response to food aversion in zebrafish

**DOI:** 10.3389/fnbeh.2013.00051

**Published:** 2013-05-22

**Authors:** Brigitte Boyer, Sylvain Ernest, Frédéric Rosa

**Affiliations:** INSERM U 1024, CNRS UMR 8197, Ecole Normale Supérieure, IBENS, Developmental BiologyParis, France

**Keywords:** zebrafish, *egr*-1, bitterness, taste aversion, consumption assay

## Abstract

As soon as zebrafish larvae start eating, they exhibit a marked aversion for bitter and acidic substances, as revealed by a consumption assay, in which fluorescent Tetrahymena serve as a feeding basis, to which various stimuli can be added. Bitter and acidic substances elicited an increase in mRNA accumulation of the immediate-early response gene *egr*-1, as revealed by *in situ* hybridization. Conversely, chemostimulants that did not induce aversion did not induce *egr*-1 response. Maximum labeling was observed in cells located in the oropharyngeal cavity and on the gill rakers. Gustatory areas of the brain were also labeled. Interestingly, when bitter tastants were repeatedly associated with food reward, zebrafish juveniles learned to ingest food in the presence of the bitter compound. After habituation, the acquisition of acceptance for bitterness was accompanied by a loss of *egr*-1 labeling. Altogether, our data indicate that *egr*-1 participates specifically in food aversion. The existence of reward-coupled changes in taste sensitivity in humans suggests that our results are relevant to situations in humans.

## Introduction

Feeding is a complex behavior that relies on the integration of different sensations produced by gustatory, olfactory, visual, and oral somatosensory stimuli. Selecting food for ingestion is a clear instance of a multisensory problem that must be solved, promptly and correctly, by any organism. For example, the food flavor is due to interactions between taste and odor modalities when they are experienced in mixtures.

In all vertebrates, the sense of taste contributes to evaluate what is palatable and what is not. Valuable nutrients such as sugars and amino acids which carry appetitive sweet and umami tastes, respectively, are ingested. In contrast, noxious and/or poisonous chemicals, such as bitter and acidic substances which carry aversive tastes are rejected. Taste stimuli produce two different forms of sensation: the purely sensory aspect which permits to recognize a few distinct taste sensations (sweet, bitter, sour, salty, and umami) on which taste memory is based and the discrimination between pleasantness or unpleasantness of the taste (the hedonic or reward vs. aversive aspect). Behavioral responses, including acceptance or rejection of the food, depend primarily on the hedonic aspect while recognition of the taste depends on purely sensory information. Taste reward (pleasure, hedonics) is obtained when the valuable food is ingested (Sewards, 2004). Conversely, gustatory aversion occurs when one ingests unpalatable taste stimuli such as bitter or acidic substances. In the case of innate pleasant tastes, the pleasure (or hedonics) is a direct consequence of the physical and chemical nature of the tastant but does not depend on previous exposure to the molecule. In contrast, taste aversion appears as a consequence of a physical malaise due to the previous ingestion of a toxic substance and is thus acquired. Accordingly, a convenient model of taste aversion relies on conditioned taste aversion (CTA), which is a learned aversion to a taste stimulus that has been followed by illness.

Small fish species such as zebrafish (*Danio rerio*) are useful models for studying sensory perception as their nervous system develops rapidly after fertilization. The zebrafish chemosensory systems of taste, olfaction and solitary chemosensory cells (SCCs) are established during the first week after fertilization, and coincide with the development of effective feeding behavior as yolk supplies diminish over the same period. The olfactory system which is the first to develop, appears anatomically and biochemically complete at 3 days after fertilization (d.p.f). The taste system relies on taste sensory cells located within taste buds abundantly distributed in the epithelia of the lips, the oropharyngeal cavity and also in the skin of the head and along the gill rakers. They first appear at about 3 d.p.f. on the lips and in the oropharyngeal cavity and at 5 d.p.f. on the outer body surface coincident with the first signs of exogenous feeding (Hansen et al., 2002; Kapsimali et al., 2011). Fish ingest and metabolize amino acids, lipids and organic acids as nutrients while they reject diets containing a bitter substance such as quinine (Lamb and Finger, 1995). Thus, there is a taste-dependent system for diet selection in fish, based on taste reward vs. gustatory aversion.

A largely shared idea is that the perception of taste pleasantness varies according to context. For example, the human perception of taste is significantly modified by other information (olfactory, visual, or somatosensory) as well as satiety, hunger and prior experience. Since the hedonic aspect of a particular food can be modified by learning, while its sensory qualities remain unchanged, it strongly suggests that there must be separate neuronal representations for the two aspects of taste, i.e., the hedonic/aversive and the purely sensory aspects. In that respect, the segregation of pathways for sensory and hedonic aspects of taste has been reported in rodents (Yamamoto and Sawa, 2000; Sewards, 2004) but not yet in fish. The modification of taste pleasantness by learning is demonstrated by the CTA assay. The assay has been widely used in rodents and demonstrated the involvement of the amygdala nuclei (for review, see Reilly and Bornovalova, 2005). Positive or negative conditioning has also been demonstrated in several fish species and particularly in zebrafish (Zippel and Domagk, 1971; Al-Imari and Gerlai, 2008; Pather and Gerlai, 2009; Gerlai, 2011; Sison and Gerlai, 2011), indicating that they have learning and memory capabilities. Moreover, taste aversion learning based on CTA has been demonstrated in fish (Gordon, 1979; Manteifel and Karolina, 1996; Martin et al., 2011). Here we demonstrate for the first time that zebrafish larvae are capable of positive associative learning, when an innate aversive taste is associated with food reward.

*Egr-1* protein is a member of a well-characterized family of immediate early gene (IEG)-encoded transcription factors playing an essential role in cellular responses that contribute to neuronal plasticity (Perez-Cadahia et al., 2011). Regarding *egr-1* induction, several studies have reported changes in its expression following a wide variety of stimuli. Interestingly, *egr-1* is essential for the formation (Jones et al., 2001) and consolidation of long-term memory (Jones et al., 2001; Bozon et al., 2002, 2003; Malkani et al., 2004). Besides its proposed role in neuronal activity, *egr-1* is also induced in a wide variety of tissues by a range of external stimuli. Its precise role in very diverse biological processes remains to be clarified. Here we show that *egr-1* induction is associated with taste aversion, suggesting that this induction may contribute to the behavioral response toward aversive food.

## Materials and methods

### Fish strains

Embryos were obtained by natural pair-wise mating in our aquaculture facility of wild-type zebrafish lines. The AB and TL strains were used equally with no obvious differences. Embryos were maintained in fish water at 28°C for ~24 h before sorting for viability. Zebrafish were staged by days post fertilization (d.p.f.) as described before (Sprague et al., 2001).

### Tetrahymena culture

Tetrahymena obtained from CCAP (Argyll, Scotland) were grown in medium containing 20 g/l proteose peptone and 2 g/l yeast extract (Sigma, St. Louis, MO). They were subcultured once a week at 1–5 dilution under sterile conditions.

### Behavioral feeding assay and data analysis

Freshly made aquarium water was used for all experimental conditions. Five d.p.f. zebrafish larvae were pooled and then split into groups of 20–30 individuals in separate wells of 12-well plastic plates. They were allowed to recover from handling and acclimated at 28°C to the new conditions for 3–4 h before the feeding assay. Tetrahymena were washed twice with aquarium water and incubated for 30 min with fluorescent calibrated beads (Polysciences, Warrington, PA). The number of Tetrahymena, their viability and fluorescent labeling was assessed under a Nikon SMZ1500 stereoscope. Preliminary experiments were conducted to determine the amount of Tetrahymena to be labeled with 1 μl of fluorescent bead solution. The various tastants (Sigma) were added to the Tetrahymena suspension prior to fish feeding. Concentrated stock solutions of the tastants were kept frozen and diluted with aquarium water immediately before use. After addition of the food suspension, the 12-well plates were returned to the 28°C incubator for an additional 30 min period. Fish were then anesthetized in 0.15 g/l MS-222 (tricaine methanesulfonate, Sigma) and photographed under a Leica upright microscope using a Nikon camera. The fluorescence intensity values were determined by integrating pixel values from images of the abdominal region using ImageJ. Background levels were obtained with the same amount of non-fluorescent food.

In the habituation protocol, larvae were subjected to a 10 min exposure to 10 mM denatonium benzoate once a day for 2 or 3 consecutive days prior to the feeding assay performed for 30 min on the next day. The experimental protocol of the training period included four different conditions that were repeated each day; (1) denatonium was added to the solution of Tetrahymena: (2) denatonium was given first, then after extensive washes, larvae were fed with Tetrahymena; (3) larvae were fed first with tetrahymena before repeated washes and addition of denatonium, (4) denatonium was given alone. As a control, larval fish were fed under the same experimental conditions with Tetrahymena alone.

For each condition, at least 20 larvae were analysed and the mean and standard deviation were calculated. For each experiment, results were expressed as the percent ratio between the experimental condition and control conditions in which larvae were fed with Tetrahymena alone. The standard deviation of the ratio was calculated using the formula:

$$\text{sd}\left\{\text{a}/\text{b}\right\}=\left(\text{a}/\text{b}\right)\sqrt{\left(\text{sd}{\left\{\text{a}\right\}}^{2}/{\text{a}}^{2}\right)+\left(\text{sd}{\left\{\text{b}\right\}}^{2}/{\text{b}}^{2}\right)}$$

Each experiment was repeated at least three times with independent batches of larvae. When appropriate, Student's *T*-Test was calculated to obtain a p value.

### *In situ* hybridization and immunohistochemistry

Whole-mount *in situ* hybridizations were performed essentially as described in the Zebrafish Book (http://zfin.org/). The construct encoding *Zegr*-1 was kindly provided by Dr. W. Norton (University of Leicester, UK). *Egr*-1 digoxigenine riboprobe was synthesized by NotI linearization and SP6 transcription for antisense probe, BamH1 and T7 for sense probe. Sense controls produced no staining and were not repeated routinely. Brain areas were identified according to the zebrafish atlas Mueller and Wullimann (2005) and to Kapsimali et al. (2007). Embryos were photographed on a Leica upright microscope using a Nikon camera. Adobe Photoshop was used to adjust brightness/contrast and mount images.

For *in situ* hybridization combined with whole-mount immunostaining, larvae were fixed in 4% paraformaldehyde at 4°C overnight prior to dehydration in 100% methanol. After rehydration in PBST (0.1% Tween 20 in PBS), larvae were subjected to proteinase K treatment (5 μg/ml) for 1 h. Samples were then incubated in hybridization buffer (50% formamide, 5X SSC, 500 μg/ml yeast tRNA, 50 μg/ml heparin, and 0.1% Tween 20 pH 6.0) containing 1 ng/μl DIG-labeled antisense RNA probe at 63°C overnight. After extensive washes, the hybridized embryos were incubated at 4°C overnight with rabbit anti-DIG Fab fragment conjugated to alkaline phosphatase (Roche, 1:5000) and mouse anti-calretinin antibodies (anti-cal2b, Swant, 1:500). After repeated washes, samples were incubated in Fast Red staining solution. When satisfactory fluorescent coloration was achieved, samples were washed with PBS-1%Triton and incubated overnight at 4°C with Alexa Fluor 488-conjugated goat-anti mouse IgG (Invitrogen, 1:500). Fluorescent images were obtained by Leica SP5 confocal microscope using 25× and 40× oil immersion objectives. Series of images were acquired at 0.4–2.5 μm intervals. ImageJ (NIH) and Adobe Photoshop were used to analyse stacks, adjust brightness/contrast and mount images.

### Semi-quantitative RT-PCR

Twenty to twenty five lower jaws of control and treated larvae were harvested in RNA later reagent (Qiagen). First strand cDNA was generated using total RNA isolated from the lower jaws using RNA Now reagent (Biogentex). RNA was quantified by spectrophotometry and all samples were adjusted to 200 ng/μl. cDNA was synthesized from 400 ng RNA using an oligo dT primer and RevertAid Premium reverse transcriptase (Fermentas). The linear amplification ranges were determined for the two primer sets: egr-1UP 5′AGTTTGATCACCTTGCTGGAG 3′ and egr-1 DO 5′GATCTCACTTTGCTGCTGTG 3′ that generate a 535 bp amplicon; trpm5 UP 5′CGGGCGATTGGGAGATATTG 3′ and trpm5 DO 5′CTGCGCTTCCTGACTCTGAC3′ that generate a 955 bp amplicon. *Egr*-1 DNA primers were designed to amplify a PCR product that spans a 705 bp intron to distinguish between genomic DNA and cDNA.

## Results

### Zebrafish larvae are sensitive to the aversive effect of bitter and acidic substances.

**Figure 1:**
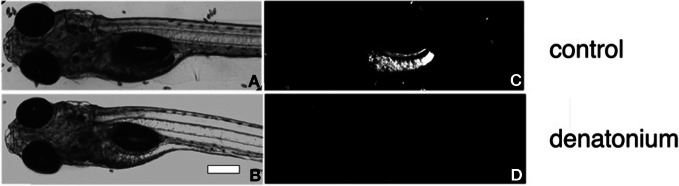
**Feeding assay of zebrafish larvae.** Bright-field micrographs of 5 d.p.f. larvae **(A,B)** corresponding to the fluorescent images **(C,D)** of the same fish fed with a solution of fluorescent Tetrahymena alone **(A,C)** or in the presence of 10 mM denatonium **(B,D)**. Heads are oriented to the left. Scale bar, 200 μm.

In order to set up an assay for the acquisition and regulation of taste aversion in fish, we have taken advantage of the fact that zebrafish larvae, at the age of only 5 d.p.f. readily prey on live Tetrahymena, by either chasing the prey or remaining stationary and sucking the prey into their mouths (Borla et al., 2002). We have set up a feeding assay using live fluorescent Tetrahymena added to plates containing 20–25 zebrafish larvae. After a 30-min delay, zebrafish were anesthetized and abdominal fluorescence was observed and photographed under an epifluorescence microscope (Figures 1A,C). Feeding assays were performed in the light at high Tetrahymena concentration. The 30 min duration of the assay prevented the complete digestion and excretion of the fluorescent preys. Under those conditions, prey consumption was not dependent on visuomotor cues, as shown by the modest impairment of feeding performances in larval fish fed in darkness (data not shown). Consistent with previous findings (Aihara et al., 2008), zebrafish larvae rejected bitter substances such as denatonium benzoate, cycloheximide, or caffeine (Figures 1B,D and Table 1). When mixed with fluorescent Tetrahymena, these substances prevented in a concentration-dependent manner the ingestion of the preys. Acids, such as acetic and citric acid, added at a concentration that did not alter the pH of the fish medium were also aversive for zebrafish larvae (Table 1).

**Table 1:** **Various prototypical tastants generate diverse feeding behaviors of zebrafish larvae**.

**Tastant**	**Food consumption (% of control^a^)**	***SD*^b^**
−	100	54
cycloheximide (5 mM)	4	4
cycloheximide (1 mM)	13	14
denatonium (10 mM)	1.4	1.8
denatonium (2 mM)	20	15
caffeine (5 mM)	1.4	1.7
glutamate (30 mM)	96	54
glutamate (30 mM) + denatonium (10 mM)	15	16
glutamate (30 mM) + denatonium (2 mM)	63	41
citric acid (2 mM)	0.5	0.4
acetic acid (2 mM)	4	3
glutamate (30 mM) + acetic acid (2 mM)	64	43

Pools of 20–25 larvae (5 d.p.f.) were fed for 30 min with a suspension of fluorescent Tetrahymena in the absence or presence of various tastants. The amount of ingested food was quantified by epifluorescence imaging and computerizing of the digital images according to the protocol described under Materials and Methods. The results are expressed as percentages of the control pool of larvae fed exclusively with fluorescent Tetrahymena. The table shows the results obtained in one typical experiment.

a Control was considered as the pool of larvae fed exclusively with the fluorescent tetrahymena solution.

b Standard deviation (SD) was calculated as mentioned under Materials and Methods.

Monosodium L-glutamate is a prototypical umami tastant which is widely recognized as an appetitive substance, even in fish (Aihara et al., 2008). When added together with fluorescent Tetrahymena, monosodium L-glutamate did not increase prey consumption, probably because the basal conditions of the assay already maximized prey ingestion. In contrast, L-glutamate was able to partially reverse the aversive effect of denatonium, as shown by the significant increase of food consumption in the presence of both substances, as compared to denatonium alone (Table 1, *p* < 0.02 and *p* < 0.001 for denatonium 10 mM and 2 mM, respectively). L-glutamate was also able to partially interfere with acids, masking their aversive effect (Table 1, *p* < 0.001). The partial recovery of a normal feeding behavior in the presence of L-glutamate suggested that the anorectic effect of bitter and acid substances was not due to their toxicity leading to fish illness. Our results are in agreement with the previous report showing that pellets flavored with bitter substances such as caffeine or quinine mixed to appetitive amino acids are more frequently eaten than pellets containing the bitter substance alone (Lamb and Finger, 1995). It remains to be determined at which level of the gustatory pathway L-glutamate masks the repulsive effect of bitter and acidic substances.

### Egr-1 immediate early gene is induced by aversive tastants

To explore the functional basis of taste-driven feeding behavior of zebrafish, analysis of the expression of *egr*-1, a member of the IEG family was performed after stimulation of larvae with various taste stimuli. The IEG products are thought to be involved in the mechanisms by which sensory stimuli trigger long-term changes and modify the neuronal response to the subsequent events (Bailey et al., 1996). *Egr*-1 (also known as *zif*268, *NGFI*-1, or *krox*-24), encoding a member of a family of inducible zinc finger transcription factors, has been implicated in neuronal changes underlying long-term synaptic plasticity and/or memory formation in a variety of memory systems (Knapska and Kaczmarek, 2004), as well as memory consolidation and reconsolidation (Renaudineau et al., 2009; Maddox and Schafe, 2011).

**Figure 2:**
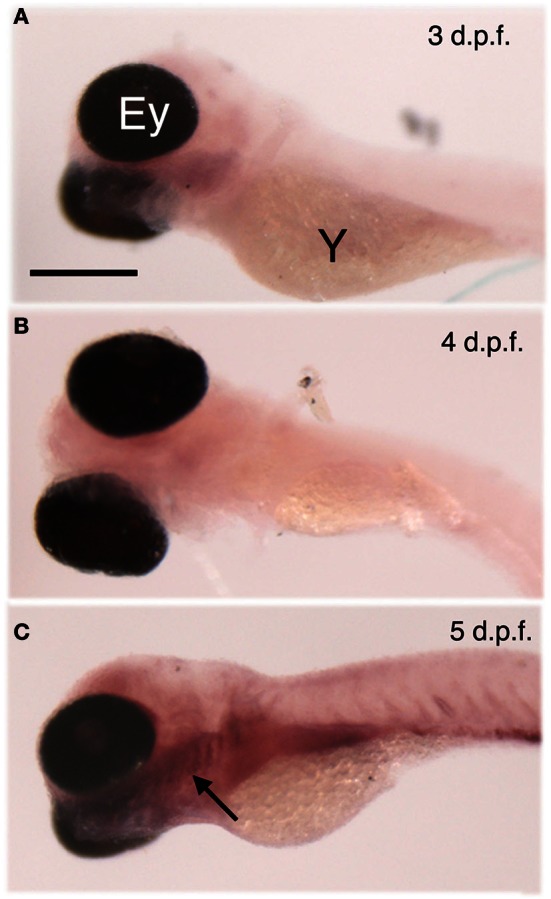
**Age-dependent *eg*r-1 induction by denatonium.** Larvae of 3 **(A)**, 4 **(B)**, and 5 **(C)** d.p.f. were incubated for 30 min with 10 mM denatonium and fixed prior to ISH with *egr*-1 specific probe. The arrow points to *egr*-1 positive-area. Ey, eye; Y, yolk. Heads are to the left. Scale bar, 100 μm.

We examined the expression of *egr*-1 mRNA by whole-mount ISH of 3, 4, and 5 d.p.f. larvae stimulated for 30 min with 10 mM denatonium benzoate, a concentration previously shown to induce a strong rejection behavior (Table 1). Whereas larvae aged 3 and 4 d.p.f. showed a very faint labeling, a strong signal was observed in the lower jaw and pharynx of 5 d.p.f larvae (Figure 2, compare **C** with **A** and **B**). The age at which specific expression was detected in stimulated larvae correlated with the development of taste and active feeding behavior. Since the olfactory system has been demonstrated to develop earlier and to be physiologically active at 4 d.p.f. (Lindsay and Vogt, 2004), denatonium is likely to induce *egr*-1 specific staining via its taste properties rather than its putative odor.

**Figure 3:**
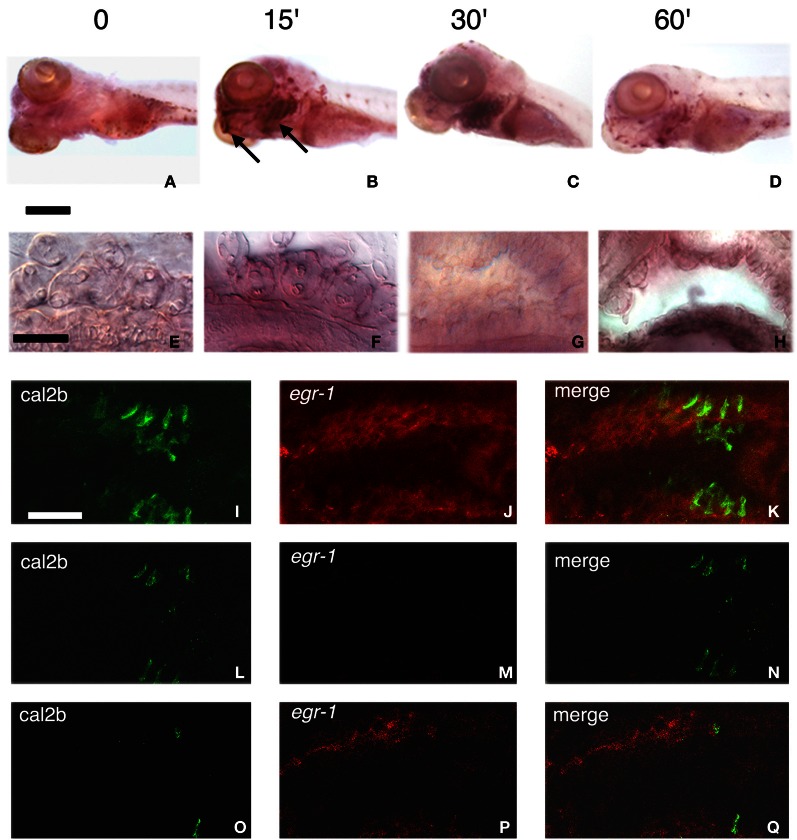
**Stimulation of *egr*-1 mRNA expression by bitterness. (A–D)** Five d.p.f. larvae unstimulated **(A)** or stimulated for 15 **(B)**, 30 **(C)**, or 60 **(D)** min with 10 mM denatonium were fixed prior to ISH with *egr*-1 specific probe. Note the strong labeling of mouth opening and gill arches (arrows), particularly visible after 15 and 30 min stimulation. Scale bar, 200 μm. **(E–H)** Microtome transverse sections of the head of non-stimulated **(E,G)** and denatonium-stimulated **(F,H)** 5 d.p.f. larvae show *egr*-1 expression in cells present at the surface of gill rakers **(E,F)** and of the oral cavity **(G,H)**. Scale bar, 50 μm. **(I–Q)** Lateral view of whole-mount larva showing two consecutive gill arches with cal2b immunostaining **(I,L,O)**, *egr*-1 specific hybridization **(J,M,P)** and the merged image of the two stains **(K,N,Q)**. **(I,J,K)** correspond to stacks of 50 images taken at 0.5 μm intervals. **(L,M,N)** correspond to the focal plan where many taste receptor cells are visible with no detectable *egr*-1 staining. **(O,P,Q)** correspond to the focal plan where *egr*-1 maximum staining is observed with a few cal2b positive taste cells.

We then sought to determine the time course of *egr*-1 stimulation of expression. Induction of *egr*-1 mRNA was obvious as early as 15 min after addition of the bitter substance, peaked after 30 min stimulation and decreased thereafter, while control non-stimulated fish exhibited only a faint staining (Figures 3A–D). Labeling in intact larvae was mostly detected in the gills where taste buds are known to be present (Hansen et al., 2002; Barreiro-Iglesias et al., 2010) (Figures 3B–D). In addition, in microtome sections of the head of denatonium-stimulated as compared to non-stimulated larvae, labeling was concentrated in superficial cells of pharyngeal arches (Figure 3F) and oropharyngeal cavity (Figure 3H). In order to examine whether denatonium-responding *egr*-1 positive cells are taste receptor cells, we performed whole-mount ISH with *egr*-1 specific probe followed by immunohistological staining of calretinin, a marker of zebrafish taste receptor cells (Kapsimali et al., 2011). The merged images (Figures 3K,N,Q) of *egr*-1 mRNA (Figures 3J,M,P) and calretinin protein (Figures 3I,L,O) specific labeling clearly show no colocalization of the two stains, indicating that denatonium-responding *egr*-1 positive cells are not differentiated taste receptor cells.

**Figure 4:**
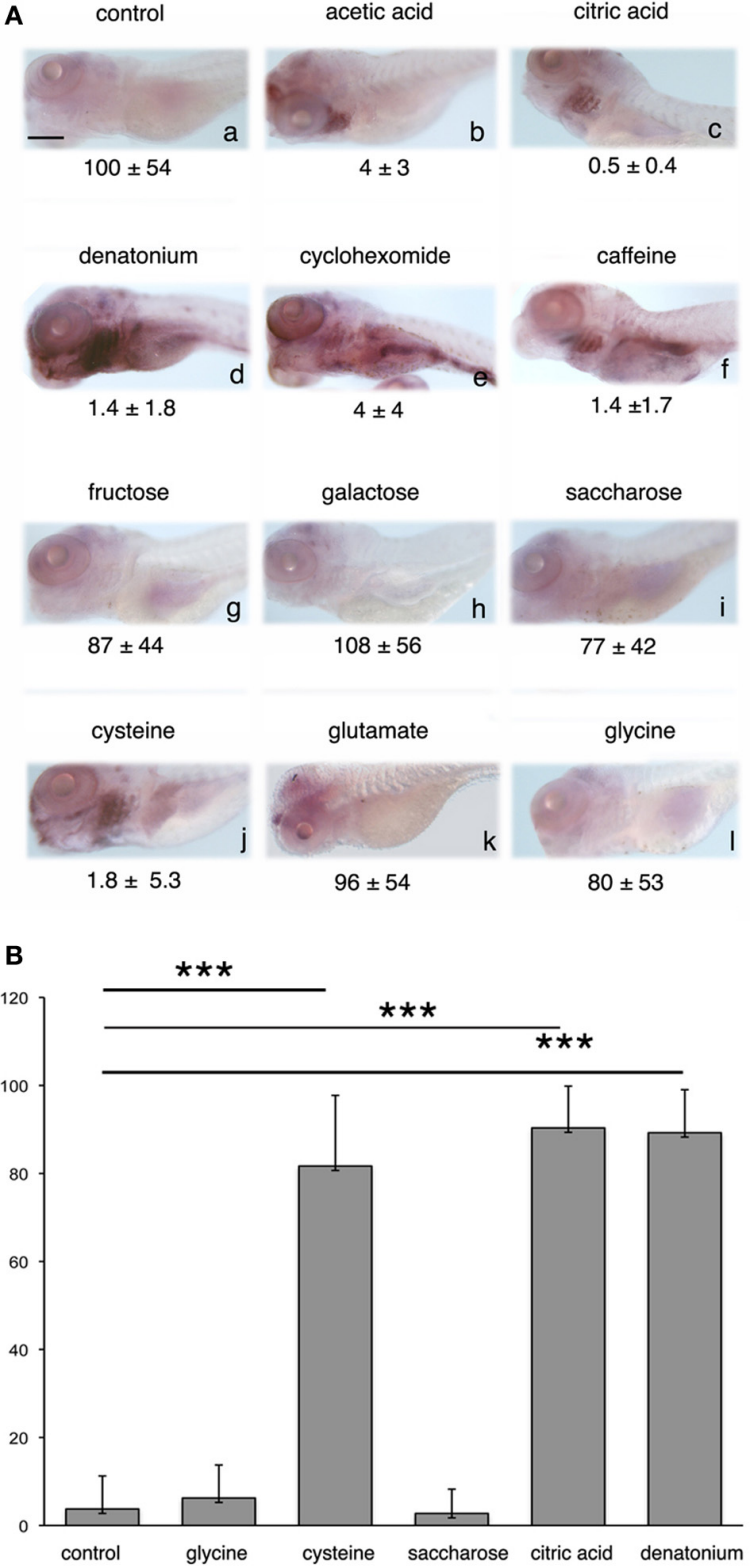
***egr*-1 expression is enhanced by aversive flavors. (A)** Five d.p.f. larvae were not stimulated **(a)** or stimulated **(b–l)** for 30 min with the tastants indicated on top of each picture before fixation and whole-mount ISH with *egr*-1 probe. Acetic acid **(b)** and citric acid **(c)** were used at 2 mM (final concentration), denatonium **(d)** was used at 10 mM, cycloheximide **(e)**, and caffeine **(f)** at 5 mM, fructose **(g)**, galactose **(h)**, saccharose **(I)**, cysteine **(J)**, glutamate **(k)**, and glycine **(l)** at 100 mM. Food consumption in the presence of each tastant is indicated below the corresponding picture. Scale bar, 100 μm. **(B)** The number of 5 d.p.f. larvae expressing *egr*-1 in the gill rakers was evaluated after 30 min stimulation with the tastants indicated, as compared to non-stimulated controls. The results were expressed as percentages of *egr*-1 positive larvae. Each bar represents at least 100 larvae scored in at least 3 independent experiments. The three asterisks indicate a *p*-value < 0.001.

We then explored which other gustatory stimuli could trigger *egr*-1 stimulation. Among the various substances tested, only those that elicit an aversive feeding behavior were observed to induce *egr*-1 expression in pharyngeal arches (Figure 4A, compare **b–f** and **j** to **g–i,k** and **l**). In contrast, sodium L-glutamate, which has been reported as hedonic for fish (Aihara et al., 2008) and can reverse the aversive effect of denatonium (our data) did not induce *egr*-1 response, as suggested by the faint labeling of gills (Figure 4Ak). Interestingly enough, cysteine stimulated larvae exhibited a strong *egr*-1 specific labeling in the gill arches (Figure 4Aj) together with a marked aversive feeding behavior. These results suggested that larvae were able to recognize amino acids and that different amino acids tested at the same concentration generated different feeding behaviors and different levels of *egr*-1 stimulation. Collectively, these data suggested also that *egr*-1 stimulation belongs to a gustatory aversive pathway.

Quantitative analysis was done by estimating the percentage of larvae exhibiting *egr*-1 specific labeling in the gill rakers after stimulation by various tastants, as compared to control unstimulated siblings (Figure 4B). It clearly showed that *egr*-1 was expressed in the gill rakers of the majority of cysteine-, denatonium-, and citric acid-stimulated larvae (*p* < 0.001 as compared to controls) whereas saccharose or glycine did not induce *egr*-1 expression.

### Food reward leads to acceptance of bitterness

It is clearly established that repeated exposure to an unpleasant taste can lead to its acceptance. In that respect, training during childhood is particularly efficient (Birch, 1998; Stein et al., 2003). We have searched for such a behavioral change in zebrafish larvae exposed to the disagreeable bitter taste of denatonium. For that purpose, larvae were subjected to a habituation period of two days (see Materials and Methods). As shown in Table 2A, 7 d.p.f. larvae trained with Tetrahymena alone did not eat Tetrahymena mixed with denatonium. In sharp contrast, when denatonium was mixed with Tetrahymena, or when denatonium was given prior to Tetrahymena during the habituation period, the 7 days old larvae ingested significantly more denatonium-containing fluorescent food (*p* < 0.002 and *p* < 0.01, respectively). They did not eat denatonium-containing Tetrahymena solution when the habituation was done with Tetrahymena followed by denatonium or with denatonium alone. This suggested that acceptance of bitterness was correlated with food reward. Larval fish habituated to denatonium ate more Tetrahymena in the presence of other bitter substances such as cycloheximide (*p* < 0.1), or in the presence of citric acid (*p* < 0.05) than untrained siblings (Table 2B), suggesting that habituation dealt with the aversive aspect of feeding and not with its specific taste.

**Table 2:** **Food reward leads to a rapid habituation to bitter taste**.

**Habituation**	**Tastant**	**Food consumption (% of control)**	***SD***
**A**			
Tetrahymena	−	100	15
Tetrahymena	denatonium	0.5	0.1
Tetrahymena + denatonium	−	97	20
Tetrahymena + denatonium	denatonium	23	4
denatonium prior to Tetrahymena	−	130	30
denatonium prior to Tetrahymena	denatonium	5	5
Tetrahymena prior to denatonium	−	95	10
Tetrahymena prior to denatonium	denatonium	4	6
denatonium	−	120	33
denatonium	denatonium	5	4
**B**			
Tetrahymena	−	100	16
Tetrahymena	denatonium	0,4	0,5
Tetrahymena + denatonium	−	102	23
Tetrahymena + denatonium	denatonium	58	38
Tetrahymena	cycloheximide	6	1
Tetrahymena + denatonium	cycloheximide	24	28
Tetrahymena	citric acid	0,3	0,2
Tetrahymena + denatonium	citric acid	32	14

A batch of 5 d.p.f. larvae was split into 5 groups. For 2 consecutive days, during a training session of 10 min, the first group received a suspension of Tetrahymena alone, the second group a mixture of Tetrahymena and denatonium at 10 mM (final concentration), the third group was given denatonium for 10 min before the Tetrahymena suspension for 10 additional min, the fourth group had the Tetrahymena suspension before denatonium and the fifth group received the denatonium solution alone. At 7 d.p.f, the feeding assay with fluorescent Tetrahymena was performed. Each group of larvae was divided into two subgroups, one receiving Tetrahymena alone while the second was fed with a mixture of Tetrahymena and denatonium at 10 mM (final concentration). The amount of ingested food was quantified by epifluorescence imaging and computerizing of the digital images according to the protocol described under Materials and Methods. The results expressed as percentages of the control pool of larvae fed exclusively with fluorescent Tetrahymena correspond to one typical experiment.

Larval fish forgot the positive association between bitterness and food reward quite rapidly. When tested 2 days after the end of a 2-day training period, they rejected denatonium almost to the same extent as untrained siblings (Table 3). This result is in agreement with a previous report showing that goldfish trained by transfer of taste preference did not recall the information over a long period (Zippel and Domagk, 1971). However, when trained over a 3-days period, zebrafish larvae retained the memory of the positive association between bitterness and food (*p* < 0.05 between untrained animals fed with Tetrahymena + denatonium as compared to trained animals), indicating that the duration of the training period influences the duration of memory.

**Table 3:** **The duration of associative memory for bitter taste depends on the duration of the training period**.

**Habituation**	**Training period**	**Tastant^a^**	**Age of larvae^b^**	**Food consumption (% of control)**	***SD***
Tetrahymena	5–6 d.p.f.	−	7	100	38
Tetrahymena	5–6 d.p.f	−	8	100	19
Tetrahymena	5–6 d.p.f	denatonium	7	2	6
Tetrahymena	5–6 d.p.f	denatonium	8	1	0, 9
Tetrahymena + denatonium	5–6 d.p.f	−	7	103	32
Tetrahymena + denatonium	5–6 d.p.f	−	8	110	50
Tetrahymena + denatonium	5–6 d.p.f	denatonium	7	27	13
Tetrahymena + denatonium	5–6 d.p.f	denatonium	8	5	3
Tetrahymena	5–7 d.p.f.	denatonium	8	3	1
Tetrahymena	5–7 d.p.f.	denatonium	9	4	2
Tetrahymena + denatonium	5–7 d.p.f.	−	8	120	36
Tetrahymena + denatonium	5–7 d.p.f.	−	9	115	44
Tetrahymena + denatonium	5–7 d.p.f.	denatonium	8	27	15
Tetrahymena + denatonium	5–7 d.p.f.	denatonium	9	21	15

A batch of 5 d.p.f. larvae was divided into 4 groups. The first group received Tetrahymena for training sessions of 10 min each for 2 consecutive days at 5 and 6 d.p.f. The second group was trained under the same conditions with a mixture of Tetrahymena and denatonium at 10 mM (final concentration). The third and fourth groups were trained for 3 consecutive days at 5, 6, and 7 d.p.f. with either Tetrahymena alone or a mixture of Tetrahymena and denatonium. At the end of the training period, each group was split into four subgroups, two of which were subjected the day after to a feeding assay using a suspension of fluorescent Tetrahymena alone or in combination with denatonium. The two others subgroups were left for 2 days prior to the feeding assay done under the same conditions. The amount of ingested food was quantified by epifluorescence imaging and computerizing of the digital images according to the protocol described under Materials and Methods. The results expressed as percentages of the control pool of larvae fed exclusively with fluorescent Tetrahymena arise from one typical experiment.

a Denatonium was used as aversive tastant during the feeding assay performed 1 or 2 days after the end of the training period.

b The age of larvae refers to that when the feeding assay was performed.

**Figure 5:**
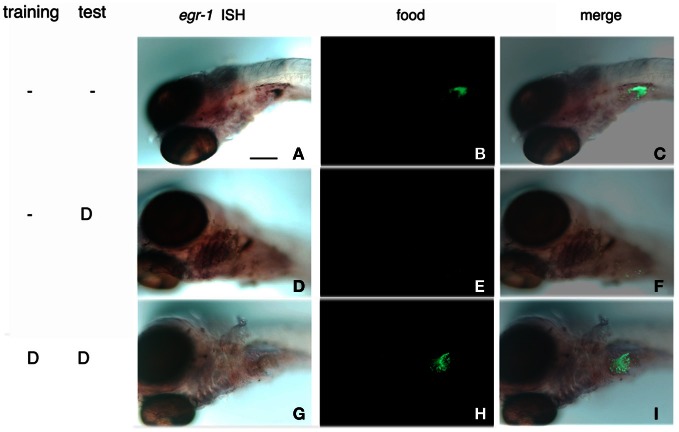
**Habituation to bitterness is accompanied by the loss of *egr*-1 stimulation of expression in gill rakers.** Zebrafish larvae were incubated for 10 min with a suspension of Tetrahymena in the absence **(A–F)** or presence **(G–I)** of 10 mM denatonium for 2 consecutive days. At 7 d.p.f., they were assayed for their ability to ingest fluorescent Tetrahymena in the absence or presence **(D–I)** of 10 mM denatonium. After fixation, the larvae were processed for whole-mount ISH with *egr*-1 probe, the fixation of which was revealed by NBT-BCIP staining **(A,D,G)**. Ingestion of fluorescent Tetrahymena was seen by epifluorescence microscopy of the abdominal region of the same larvae **(B,E,H)**. **(C,F,I)** are the merged images of the phase contrast and epifluorescence pictures. **(D)** stands for denatonium.

Interestingly enough, acceptance of bitterness, demonstrated by the ingestion of fluorescent Tetrahymena in the presence of denatonium (Figure 5, compare **H** with **E**), was accompanied by the loss of *egr*-1 stimulation in pharyngeal arch responding cells (Figure 5, compare **G** with **D**). This result reinforced the idea that *egr*-1 plays a role in a gustative aversive pathway.

**Figure 6:**
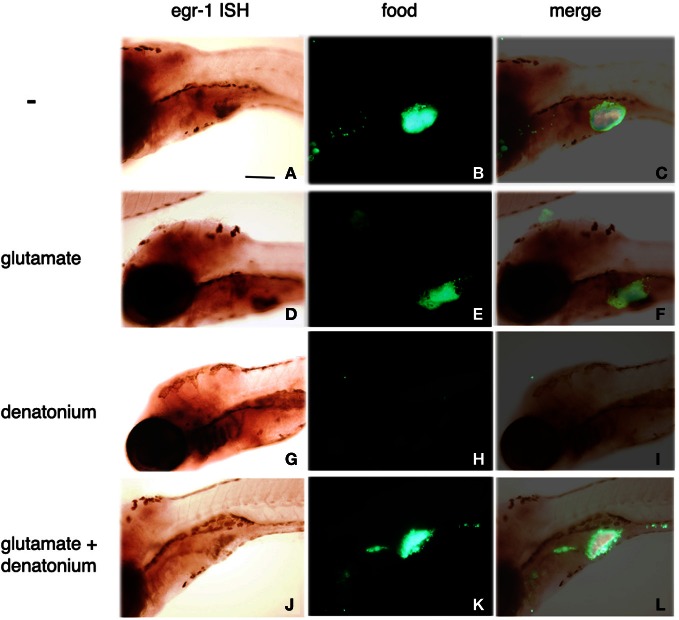
**Sodium glutamate-induced acceptance of denatonium is accompanied by the lack of *egr*-1 expression.** Five d.p.f. larvae were incubated for 30 min with a suspension of fluorescent Tetrahymena in the absence of tastant **(A–C)**, in the presence of 30 mM sodium glutamate **(D–F)**, 10 mM denatonium benzoate **(G–I)**, or a mixture of glutamate and denatonium **(J–L)** before fixation and whole-mount ISH. *Egr*-1 probe fixation was specifically revealed by NBT-BCIP staining **(A,D,G,J)**. Ingestion of fluorescent Tetrahymena was monitored by epifluorescence microscopy of the abdominal region of the same larvae **(B,E,H,K)**. **(C,F,I,L)** are the merged images of the phase contrast and epifluorescence pictures.

To confirm the implication of *egr*-1 in taste aversion, 5 d.p.f. larvae were fed with fluorescent Tetrahymena in the absence or presence of glutamate, denatonium or both. As already mentioned, glutamate was able to revert the aversive effects of denatonium, leading to the ingestion of fluorescent preys incubated with a mixture of denatonium and glutamate (Figure 6, compare **K** with **H**). Under these conditions, *egr*-1 was not expressed whereas it was evidenced in the gill rakers of larvae fed with denatonium and fluorescent Tetrahymena (Figure 6, compare **J** and **G**). These results strengthen the idea that *egr*-1 is specifically implicated in the taste aversion response.

**Figure 7:**
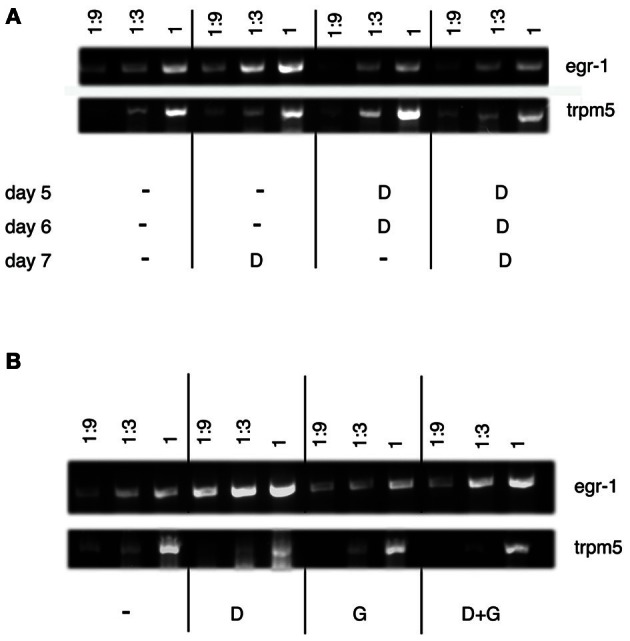
**Semi-quantitative RT-PCR analysis of *egr*-1 expression in zebrafish larvae. (A)** RT-PCR was performed on lower jaws of zebrafish larvae (7 d.p.f.) that were fed with Tetrahymena alone (−) or together with 10 mM denatonium (D) for 3 consecutive days, according to the protocol of habituation. Serial dilutions (1, 1:3, and 1:9) of cDNAs were amplified by PCR. RT-PCR shows a semi-quantitative increase in *egr*-1 mRNA expression in larvae exposed to denatonium for the first time on day 7 as compared to the absence of increase of *egr*-1 mRNA expression in larvae habituated to denatonium for 3 consecutive days. *TrpM5* was used as a control. **(B)** RT-PCR was performed on lower jaws of zebrafish larvae (5 d.p.f.) that were fed with Tetrahymena alone (−) or in the presence of 10 mM denatonium (D), 30 mM sodium glutamate (G), or a mixture of both (D + G) for 30 min. Serial dilutions (1, 1:3, and 1:9) of cDNAs were amplified by PCR to show a marked semi-quantitative increase in *egr*-1 mRNA expression in larvae exposed to denatonium as compared to animals treated with glutamate or glutamate and denatonium. *TrpM5* was used as a control.

RT-PCR analysis of *egr*-1 expression under conditions leading to acceptance of bitterness was done in order to confirm the ISH data (Figure 7). Serial dilutions of cDNA reverse-transcribed from RNAs extracted from lower jaws of larvae were subjected to PCR with *egr*-1 and *TrpM5* specific primers. The expression pattern of *TrpM5* is limited to specialized cell types (Sprous and Palmer, 2010). *TrpM5* is expressed by type II taste cells abundantly found in the lower jaw of zebrafish and cells of the gastrointestinal tract. It was therefore used as a regional marker to ensure that contamination by other tissues was similar under each condition. As shown in Figure 7A, habituation to denatonium led to a marked decrease of the *egr*-1 specific signal as compared to 7 d.p.f. larvae exposed to denatonium for the first time. Similarly, addition of glutamate to denatonium induced a reduction in the *egr*-1 signal obtained with denatonium alone (Figure 7B). It should be noted that glutamate was not able by itself to generate an increase in *egr*-1 specific PCR products, corroborating the ISH data.

### Denatonium-induced egr-1 expression in the brain is counteracted by habituation to bitterness

**Figure 8:**
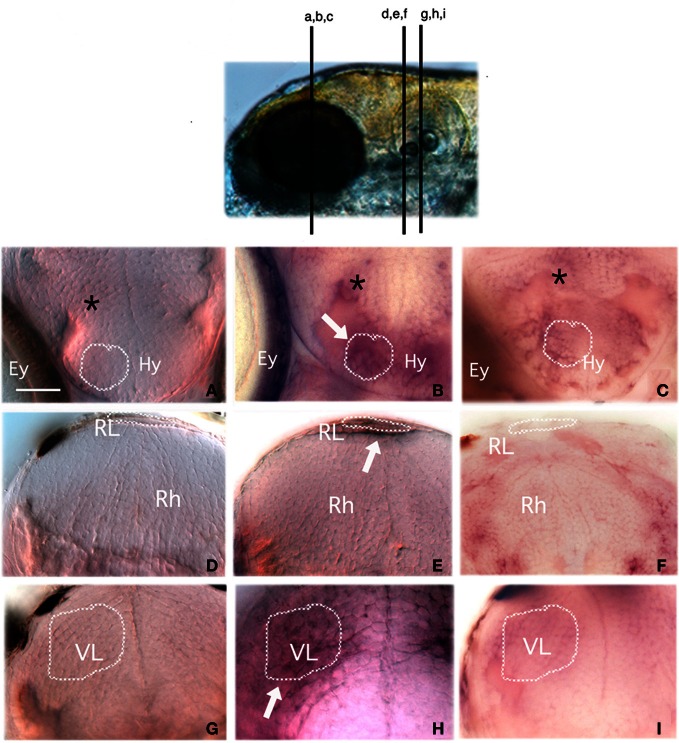
***Egr*-1 is expressed in gustatory brain areas.** Larvae non-stimulated **(A,D,G)** or stimulated with 10 mM denatonium for 30 min without prior habituation to denatonium **(B,E,H)** or with repeated exposures for 2 consecutive days to denatonium **(C,F,I)** were fixed and subjected to ISH with an *egr*-1 specific probe. They were then embedded in agarose before microtome sectioning through the whole head. Transverse sections were harvested on glass slides and mounted for microscopic observation. **(A,B,C)** Sections through the hypothalamic region. Stimulated larva **(B)** exhibits labeling in hypothalamic cells (arrow) and cells located dorsal to the hypothalamus (asterisk) not visible in control larva **(A)** or in larva habituated to denatonium **(C)**. Ey, eye; Hy, hypothalamus region **(D,E,F)** Sections through the rhombencephaphalic region (Rh) in which the rhombencephalic lip (RL) of stimulated larva is labeled (arrow) as opposed to control animal. **(G,H,I)** Sections through the vagal lobe (VL). The stimulated larva exhibits a strong staining contrasting with the absence of *egr*-1 expression in control and habituated larvae. Dark spots visible at the periphery of the brain in **(D,G,I)** correspond to melanocytes.

We also sought to determine whether *egr*-1 expression could be stimulated by bitterness in some parts of the taste sensory system and, in particular, in the brain. Microtome sectioning of control and denatonium-stimulated larval brains was performed to identify the anatomical structures recognized by the *egr*-1 probe (Figure 8). The hypothalamus, the rhombic lip and the vagal lobe displayed *egr*-1-specific expression in denatonium-stimulated animals (Figures 8B,E,H) contrasting with the lack of labeling in controls (Figures 8A,D,G). Primary gustatory centers of fish have been localized in the rhombencephalon and vagal lobe, secondary and tertiary gustatory nuclei have been found in the posterior area of thalamus as well as in the dorsal hypothalamus (Folgueira et al., 2003; Ikenaga et al., 2009). Additional *egr*-1 expression was seen at cells dorsal to the hypothalamus of denatonium-stimulated larvae (Figure 8B, asterisk), which may correspond to gustatory cells of the preglomerular complex. Altogether, our data indicated that denatonium benzoate elicited a strong *egr*-1 response in brain structures involved in processing gustatory information. Furthermore, in denatonium-habituated larvae, no labeling of the hypothalamus, rhombic lip and vagal lobe was observed (Figures 8C,F,I), suggesting that *egr*-1 positive brain areas participate in aversive feeding behavior.

## Discussion

In this article we demonstrate for the first time taste-induced stimulation of the IEG *egr*-1 in gill arches, oropharyngeal cavity and in brain areas participating in the gustatory information. The specificity of the activation was demonstrated by the lack of taste-dependent activation of *egr2-b*, another member of the *egr* family of transcription factors (datat not shown). *Egr*-1 mRNA expression was evidenced in superficial cells of the oropharyngeal cavity and pharyngeal arches in the vicinity of taste receptor cells. The precise nature of *egr*-1 expressing cells remains to be clarified. However, it is plausible that they represent the epithelial contribution to these anatomical structures. It remains to elucidate the pathway(s) elicited by an aversive taste stimulus leading to *egr*-1 expression in brain areas devoted to gustatory processing and oropharyngeal cells. In brain neurons, *egr*-1 expression may result from the signal emanating from peripheral chemosensory organs. Alternatively, it could arise from the stimulation of bitter taste receptors shown recently to be expressed in rat brain cells (Singh et al., 2011). Additionally, the role of *egr*-1 in these cells would deserve further investigation. Interestingly, *egr*-1 stimulation was exclusively elicited by aversive tastants such as bitter and acidic substances, suggesting that it may take part in a signaling pathway involved in aversion. It is tempting to postulate that *egr*-1 induction operates in the response aimed to protect cells against the deleterious effects of toxics.

We set up a behavioral feeding assay designed to study the developmental regulation of feeding aversion. Aversion appeared early, at 5 d.p.f. in naïve animals that had never been exposed to repelling tastes. Therefore, an aversive behavior was established immediately after the first exposure to distasteful food, strongly suggesting that this was an innate behavior, although we cannot rule out that larvae have enough time to learn to develop aversion. It is most likely that, as already described in other species (Lamb and Finger, 1995), when zebrafish takes the prey in its mouth, the aversive tastant prevents it to swallow and leads to spitting out the prey. The threshold for denatonium and citric acid aversion determined in this study was high, since the maximal effect was observed for 5 mM denatonium and 2 mM citric acid and decreased for lower concentrations. However, the behavioral threshold of zebrafish for denatonium and citric acid is probably lower than we were able to demonstrate. As evidenced by their high consumption rate, the Tetrahymena used in our feeding assay are likely not tasteless to zebrafish and they contain many appetitive amino acids that may interfere with the unpleasantness of denatonium or citric acid, in contrast to the neutral carrier used in previous studies (Aihara et al., 2008). Accordingly, we observed that addition of the appetitive sodium glutamate counterbalanced the aversive effect of denatonium or citric acid. However, our assay provides the advantage to measure directly and simply a threshold of sensitivity, and to combine different tastants.

Although sensitivity of fish taste cells to bitter and umami (amino acid) diets has been largely reported (Valentincic and Caprio, 1994; Lamb and Finger, 1995; Kohbara and Caprio, 1996; Borla et al., 2002; Oike et al., 2007), there is few evidence if any that fish react to sugars (Hashiguchi et al., 2007). This may explain the absence of *egr*-1 expression after exposure to sugars. On the other hand, the absence of a behavioral feeding response to a sugar-rich diet may indicate that the sweet taste is neutral, neither aversive nor attractive. As already mentioned, most amino acids are appetitive for fish but fish react with a variety of behaviors to individual amino acids. In that respect, cysteine appears as an aversive taste, eliciting a marked decrease of Tetrahymena ingestion when given at a concentration above 10 mM. Accordingly, zebrafish juveniles as well as young adults react to the amino acid L-cysteine with an aversive behavioral response, moving away from the place where L-cysteine stimulus takes place (Vitebsky et al., 2005). It should be noted that cysteine, as other amino acids, may act as an olfactory (Lindsay and Vogt, 2004) as well as a taste stimulus. The strong *egr*-1 labeling of the gills observed after stimulation with cysteine may thus be correlated with taste or odor-driven aversion for the amino acid.

*Egr*-1 induction appears to be restricted to the hedonic (aversive vs. appetitive) aspects of taste, first because it is strictly associated with the rejection of food upon aversive compounds (bitter, acid) exposure and second because the presence of glutamate can relieve both the aversion and the expression of *egr*-1. This is consistent with the existence, within the rodent gustatory system, of separate sensory and hedonic (reward vs. aversion) representations in the primary structures in which processing of the gustatory stimuli occurs (Sewards, 2004; Knapska et al., 2006). It remains to demonstrate, however that similar pathways exist in zebrafish. Although taste transduction is being actively studied, signaling mediating the hedonic taste response is poorly understood and *egr*-1 induction thus represents a convenient entry point to approach this problem.

The finding that zebrafish larvae, which spontaneously dislike bitter substances are amenable to ingest bitter food after training indicates that zebrafish larvae have learning and memory capabilities. This is in line with numerous reports (Zippel and Domagk, 1971; Al-Imari and Gerlai, 2008; Sumbre et al., 2008; Pather and Gerlai, 2009; Gomez-Laplaza and Gerlai, 2010; Sison and Gerlai, 2010; Gerlai, 2011; Sison and Gerlai, 2011). In mammals, one learning process related to taste memory is taste aversion learning that consists in avoiding the ingestion of substances previously associated with visceral discomfort. Taste aversion learning in fish has also been demonstrated (Mackay, 1974; Martin et al., 2011) and the role of the dorsomedial pallium, which emerges as the homolog of the amygdala in mammals has been proposed in this process (Martin et al., 2011). However, no taste aversion conditioning has been evidenced in zebrafish. Here we demonstrate instead a positive association learning involving a previously disliked taste associated with food reward. Whether or not the dorsomedial pallium plays a role in establishing and maintaining this memory remains to be explored. Accordingly, in mammals, the amygdala has been shown to influence appetitive-driven behaviors (Savage and Ramos, 2009). Learning capacity has been demonstrated in zebrafish larvae as well (Wolman et al., 2011; Valente et al., 2012). Our results showing that long-term memory of a positive association between food reward and bitterness is not fully established in zebrafish larvae are in agreement with previous observations (Zippel and Domagk, 1971; Valente et al., 2012). The fact that *egr*-1 stimulation, which discriminates between aversive and appetitive substances is abrogated after habituation to bitterness reinforces the idea that *egr*-1 is involved in a hedonic pathway. It is plausible that *egr*-1 expression, previously shown to be implicated in cognitive processes is related to the learning process of aversive tastes, as shown in training assays of adult rats, where the observed increase in *egr*-1/zif238 was associated with the acquisition phase of the training i.e., following one session of training procedure but not with the performance of the already well-trained task (Knapska and Kaczmarek, 2004). Feeding behavior of mutant zebrafish larvae lacking *egr*-1 expression is under current investigation. These experiments should help deciphering whether *egr*-1 participates actively in establishing the memory of aversive tastes.

The mechanisms whereby training can change an aversive behavior into a hedonic one are still unknown. It is likely that they involve brain plasticity, as they need repeated training sessions over several days to be successful. Whether or not they are identical to those operating during glutamate-driven acceptance of bitterness is another yet unsolved question. As reported recently, glutamate may activate glutamatergic synaptic receptors on mouse taste bud presynaptic cells, leading to the secretion of 5-HT and inhibition of taste-evoked ATP secretion (Huang et al., 2012). It is therefore tempting to postulate that addition of glutamate to bitter food may have two complementary roles in zebrafish: on the one hand, glutamate may act, as described in mouse, as an efferent transmitter that inhibits bitter-induced aversion signaling by reducing bitter-induced ATP secretion, leading to a marked decrease of *egr*-1 induction. On the other hand, glutamate may directly bind glutamate receptors on cognate receptor taste cells, eliciting an umami-evoked taste signaling pathway.

Altogether, our data provide cues to explore how innate aversive and hedonic tastes are established and how they can be changed by learning and experience.

### Conflict of interest statement

The authors declare that the research was conducted in the absence of any commercial or financial relationships that could be construed as a potential conflict of interest.
